# Comparison of human brain metabolite levels using 1H MRS at 1.5T and 3.0T

**DOI:** 10.1590/S1980-57642013DN70200013

**Published:** 2013

**Authors:** Fernando Fernandes Paiva, Maria Concepcion Garcia Otaduy, Ricardo de Oliveira-Souza, Jorge Moll, Ivanei Edson Bramati, Luciane Oliveira, Andrea Silveira de Souza, Fernanda Tovar-Moll

**Affiliations:** 1 PhD, Magnetic Resonance Imaging and In Vivo Spectroscopy Center (CIERMag), Physics Institute of São Carlos, University of São Paulo, São Carlos SP, Brazil.; 2 PhD, Magnetic Resonance Department, LIM44, InRad-Hospital das Clínicas, Faculty of Medicine of the University of São Paulo, São Paulo SP, Brazil.; 3 PhD, Gaffreé e Guinle University Hospital, University of the State of Rio de Janeiro, Rio de Janeiro RJ, Brazil. D'Or Institute for Research and Education (IDOR), Rio de Janeiro RJ, Brazil.; 4 PhD, D'Or Institute for Research and Education (IDOR), Rio de Janeiro RJ, Brazil.; 5 D'Or Institute for Research and Education (IDOR), Rio de Janeiro RJ, Brazil.; 6 MD, D'Or Institute for Research and Education (IDOR), Rio de Janeiro RJ, Brazil.; 7 PhD, D'Or Institute for Research and Education (IDOR), Rio de Janeiro RJ, Brazil. Biomedical Sciences Institute, Federal University of Rio de Janeiro, Rio de Janeiro RJ, Brazil.

**Keywords:** brain, magnetic resonance spectroscopy, 1.5T, 3.0T

## Abstract

Proton magnetic resonance spectroscopy (MRS) of the human brain has proven to be a useful technique in several neurological and psychiatric disorders and benefits from higher field scanners as signal intensity and spectral resolution are proportional to the magnetic field strength.

**OBJECTIVE:**

To investigate the effects of the magnetic field on the measurement of brain metabolites in a typical routine clinical setting.

**METHODS:**

Single voxel spectra were acquired from the posterior cingulate cortex in 26 healthy subjects. Each subject was scanned consecutively at 1.5T and 3.0T in a randomly distributed order.

**RESULTS:**

SNR and peak width improvements were observed at higher fields. However, SNR improvement was lower than the theoretical two-fold improvement. Other than the values obtained for creatine (Cre) and myo-Inositol (mI), which were both higher at 3.0T, all metabolite concentrations obtained were roughly the same at both field strengths. All the metabolite concentrations were estimated with a Cramer Rao lower bounds (CRLB) lower than 15% of the calculated concentrations.

**CONCLUSIONS:**

Even though the present study supports the expected benefits of higher field strength for MRS, there are several factors that can lead to different quantitative results when comparing 1.5T to 3.0T MRS. Future comparative studies are necessary to refine the metabolite thresholds for early detection and quantification of distinct neurological and psychiatric disorders using 3.0T MRS.

## Abstract (pt)

Espectroscopia de prótons por ressonância magnética (MRS) tem se mostrado uma técnica bastante útil em diversas doenças neurológicas e psiquiátricas. A utilização de sistemas de mais alto campo magnético favorece essa técnica uma vez que a intensidade do sinal e a resolução espectral são proporcionais à intensidade do campo.

**OBJETIVO:**

Avaliar o efeito do campo magnético sobre a medida dos níveis dos metabólitos cerebrais em uma típica rotina clínica.

**MÉTODOS:**

Os dados foram obtidos em 26 indivíduos saudáveis nos sistemas de 1.5T e 3.0T. As aquisições foram feitas sequencialmente e a ordem foi distribuida randomicamente.

**RESULTADOS:**

Foram observadas melhoras na relação sinal-ruído (SNR) e na largura de linha dos picos nos dados obtidos em campo maior. No entanto, a melhoria na SNR foi menor que o esperado teoricamente que seria o dobro da obtida em 1.5T. Exceto pelos valores obtidos para creatina e mio-inositol, que foram maiores em 3.0T, todas as concentrações de metabólitos obtidas foram aproximadamente a mesmo em ambos os campos. Todas as concentrações de metabólitos foram estimadas com *Cramer Rao lower bounds* (CRLB) inferior a 15% das concentrações calculadas.

**CONCLUSÕES:**

Apesar de o presente estudo dar suporte aos benefícios gerados pelo aumento do campo para a técnica de MRS, existem fatores que podem levar a diferentes resultados quantitativos quando se compara espectroscopia em 1.5T e 3.0T. Estudos comparativos serão necessários para refinar os limiares dos níveis de metabólitos para melhorar a acurácia da detecção de doenças neurológicas utilizando espectroscopia em 3.0T.

## INTRODUCTION

Proton magnetic resonance spectroscopy (MRS) has proven to be a useful non-invasive technique to obtain information regarding the normal and abnormal neurochemistry of the human brain.^1,2^ In some clinical settings, MRS may show early metabolic changes in apparently anatomically-normal tissue.^3^ MRS benefits from higher field scanners because signal intensity and spectral resolution (chemical shift) are theoretically proportional to the strength of the magnetic field.^4,5^ Another consequence is the increased J-coupling splitting. For example, the metabolite *myo*-Inositol (mI) is represented as a single peak at 3.56 ppm at 1.5T, while at 3.0T it appears to be split mainly into two peaks at 3.56 and 3.64 ppm, making its visual detection and quantification harder. Moreover, susceptibility effects are stronger at higher field strengths, resulting in larger peak linewidths. Transverse relaxation times (T2) also tend to decrease at higher fields, resulting in lower metabolite signals for a given echo time (TE) when compared to lower field strengths. These higher-field effects may have some clinical implications insofar altered levels of mI are associated with prevalent neurological disorders, such as Alzheimer's disease.

An accurate clinical interpretation of individual spectra requires the knowledge of the normal range of relative metabolite levels (or absolute concentrations), as well as an understanding of how the measured values depend on different aspects, such as patient age, region of interest, metabolic conditions, specific MRS technique and field strength. Even though some quantitative studies have been reported at different field strengths,^6-9^ most comparisons between the widespread 1.5T and 3.0T systems, which are becoming increasingly common in clinical settings, have focused on the evaluation of SNR and spectral resolution at higher fields.^10^ However, these studies have not systematically compared the metabolite concentrations and ratio estimates across different field strengths. Thus, quantitative comparisons between both field strengths are needed in order to establish reference data at 3.0T as well as to determine if the normal ranges previously established for 1.5T can be directly adopted in any system. In the present study, metabolite levels at 1.5T and 3.0T were assessed in healthy volunteers and the influence of field strength on the measured values, and on calculated metabolite ratios used for diagnostic purposes, were evaluated.

## METHODS

**1H MRS Methods.** Healthy adult volunteers (N=26, seven males and nineteen females, mean age 53±22 years) were scanned under an Institutional Review Board (IRB)-approved protocol on a 3.0T Philips Achieva system equipped with gradients capable of 80 mT/m amplitude and 200 mT/m/ms slew rate and on a 1.5T Philips Gyroscan system equipped with gradients capable of 23 mT/m amplitude and 105 mT/m/ms slew rate (Philips Medical Systems, The Netherlands). All subjects were free of neurological and neuropsychiatric disorders. A standard transmit body coil and an eight-channel receive-only head coil were used for data acquisition in both systems. Each subject was scanned consecutively at 1.5T and 3.0T in a randomly distributed order, such that half of the volunteers were scanned initially on the 1.5T system and the other half on the 3.0T system.

**Figure 1:**
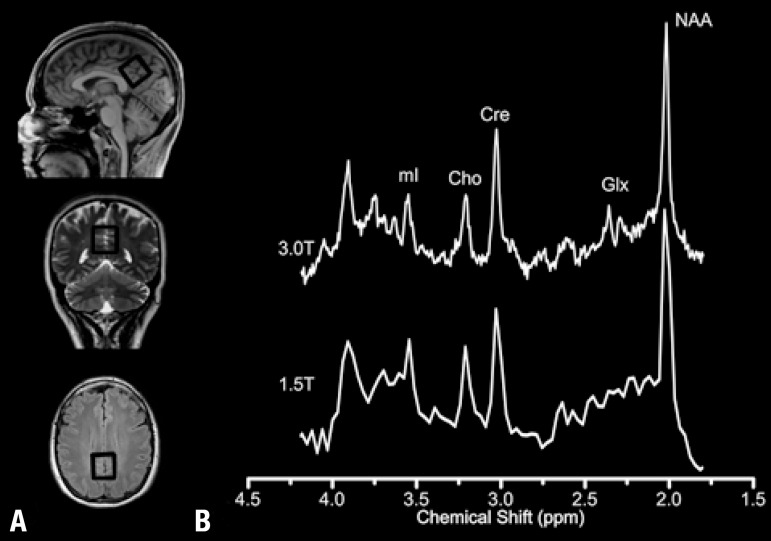
[A] Schematic representation of the voxel location used for localized MRS data acquisition, which is, due to chemical shift effects, a representation of the voxel location of the NAA signal. [B] Typical spectra obtained from a representative volunteer at 1.5T and 3.0T showing the common brain metabolites.

At both field strengths, single voxel spectroscopy was performed using a point-resolved spectroscopy sequence (PRESS). Before obtaining the spectra, automatic shimming and water suppression were conducted by the scanner. At 1.5T, spectra were acquired using the following parameters: TE=31 ms, TR=1500 ms, 512 spectral points and 1 kHz receiver bandwidth. At 3.0T, a TE=31 ms, TR=2000 ms, 2048 spectral points and 2 kHz receiver bandwidth was used. The repetition time was optimized at both field strengths for optimal signal-to-noise ratio (SNR) for the metabolites of interest. For each spectrum, a total of 128 measurements were averaged at 1.5T and 96 measurements at 3.0T resulting in the same acquisition time at both field strengths. In both cases, a voxel size of 25 x 25 x 25 mm3 positioned using a T1- and a T2-weigthed scout image at the posterior cingulate gyrus and aligned according to the parieto-occipital sulcus was used, as illustrated in Figure 1A.

**Data analysis and statistics.** All spectroscopic data were processed using LCModel.^11^ An automatic adjust of the phase and eddy current correction was applied to all spectra. Relative metabolite concentrations and their uncertainties were estimated by fitting the spectrum to a basis set of spectra acquired from individual metabolites in solution and referencing to the unsuppressed water peak, and are expressed in institutional units. Out of the basis set of spectra, a few metabolites and metabolite combinations were selected for further analysis: *N*-acetylaspartate and other *N*-acetyl-containing compounds (NAA), glutamine and glutamate (Glx), creatine and phosphocreatine (Cre), choline-containing compounds (Cho) and *myo*-Inositol (mI).

Significant differences across the concentrations of the metabolites, accuracy of the estimation through the analysis of the standard deviation of the estimated metabolite, SNR, and full-width at half maximum (FWHM) obtained at both field strengths, were tested using a non-parametric analysis (Wilcoxon signed-rank test) performed with SPSS 16 (SPSS Inc., Chicago, IL, USA). All data are expressed as mean and standard deviation and results were considered statistically significant when p<0.05.

## RESULTS

Figure 1A shows the voxel location used for data acquisition, which is, given chemical shift effects, a representation of the voxel location for the NAA signal. Figure 1B shows typical spectra obtained from a representative volunteer at 1.5T and 3.0T. Results show a better spectral resolution and SNR at 3.0T. This can be better visualized for instance in the Glx region of the spectra where better resolved peaks at 3.0T are evident compared to 1.5T.

As expected, the averaged calculated SNR at 3.0T (23±6) was significantly higher than the calculated value for 1.5T (15±4, p<0.0001). The linewidths were also statistically different at the two field strengths (3.1±0.7Hz at 1.5T and 5.6±0.9 Hz at 3.0T, p<0.05).

**Figure 2:**
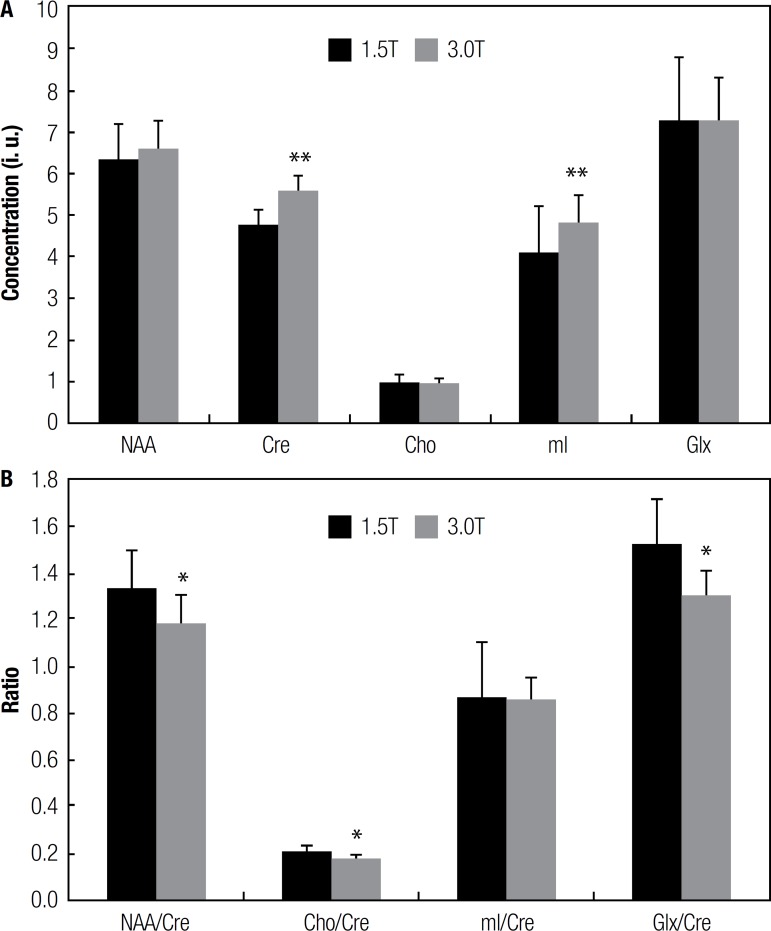
[A] Mean concentrations (in institutional units) of the analyzed metabolites obtained at 1.5T and 3.0T; and [B] their respective ratios related to Cre signal. Values are represented by mean and standard deviation (N=26, ^*^p<0.05, and ^**^p<0.005).

Figure 2A shows the average concentration of the analyzed metabolites. All the metabolite concentrations were estimated with a Cramer Rao lower bounds (CRLB) lower than 15% of the calculated concentrations and the mean CRLB values obtained at 3.0T (2.8±0.5%, 2.3±0.5%, 4.4±0.8%, 5.2±0.9%, 9.5±2.0% for NAA, Cre, Cho, mI and Glx, respectively) were statistically lower than their respective values at 1.5T (6.7±1.4%, 7.2±1.9%, 10.3±2.4%, 9.5±2.0%, 14.8±2.9% for NAA, Cre, Cho, mI and Glx, respectively, p<0.001) for all metabolites. The mean concentration obtained for NAA (6.3±0.8), Glx (7.2±1.5), and Cho (1.0±0.2) at 1.5T were not statistically different from the values obtained at 3.0T (6.6±0.7, 7.3±1.0, 0.9±0.1, respectively). However, the values obtained for Cre and mI at 1.5T (4.8±0.4and 4.1±1.1, respectively) were both lower than their respective values at 3.0T (5.6±0.3 and 4.8±0.7, respectively, p<0.005).

Figure 2B shows the average metabolite ratios for all volunteers. The ratios NAA/Cre, Cho/Cre, and Glx/Cre were lower at 3.0T (1.19±0.12, 0.17±0.02and 1.31±0.19, respectively) compared to their equivalent at 1.5T (1.33±0.16, 0.20±0.03and 1.52±0.29, respectively, p<0.05). On the other hand, the mI/Cre ratio obtained at 1.5T (0.86±0.24) was not statistically different to the value found at 3.0T (0.86±0.09).

## DISCUSSION

In the current study, brain metabolites detected at 1.5T and 3.0T in the same subject were analyzed. As expected, when compared to the values obtained at 1.5T, spectra obtained at 3.0T had higher SNR. However, the 53% increase found is well below the theoretically predicted 100% improvement. The theoretical linear increase would be expected by assuming constancy of [1] the noise generated by the system, [2] RF penetration effects, [3] T1 and T2 relaxation times, none of which is actually true.^12-16^ Furthermore, an increase in the line-widths of the metabolites at 3.0T was found that can partially counteract the SNR improvement associated with higher field strength. The average value obtained at 1.5T was 3.1 Hz, while at 3.0T the value obtained was 5.6 Hz. This increase is in agreement with previously published data,^10^ but is slightly less than the two-fold increase predicted by the theoretical relation of susceptibility effects being proportional to field strength. This might be related to the different shimming capabilities of the systems used, since only the 3.0T scanner was equipped with second order shimming.

Except for the values obtained for mI/Cre, all other metabolite ratios were smaller at 3.0T. The lower values for NAA/Cre and Cho/Cre at 3.0T are likely to be due to differences in transversal relaxation times for T2 at both field strengths. Spectra were acquired using a TE of 31ms and a TR of 1500/2000ms, which means that they are both T2- and T1-weighted. Thus, the signal intensities are related not only to metabolite concentration, but also to its relaxation properties. Consequently, metabolites with shorter T2 and longer T1 present lower signals, since the present data were not corrected for relaxation effects.

Based on the literature, expected relaxation effects can be estimated. For the cingulate gyrus at 1.5T, a T2 of 351, 336 and 188 ms for Cho, NAA and Cre, respectively, was previously reported,^17^ whereas T2 for gray matter at 3.0T was significantly shorter: 209, 216 and 131 ms for Cho, NAA and Cre, respectively.^18^ Thus, the shortening ratio of T2 at a higher field is different for each metabolite, which could explain the different metabolite ratios obtained at 3.0T compared to 1.5T. Furthermore, absolute metabolite concentrations were obtained by comparing metabolite signal intensity to water signal of an unsuppressed reference scan. Hence, in the evaluation of these absolute values the manner in which T2 and T1 of water changes with increasing field has to be taken into account. In the literature, there are reports of T2 of water brain tissue of around 107 ms at 1.5T and around 60 ms at 3.0T.^19,20^ T2 of cerebral spinal fluid (CSF) is much longer where values greater than 1s at 1.5T, and around 500 ms at 3.0T, having been reported.^20,21^ Thus, the significant higher absolute values for Cre and mI at 3.0T and a trend toward higher values for NAA at 3.0T might be related to a stronger T2 shortening of the water signal at 3.0T as compared to these metabolites. Changes in T1 can also partially contribute to the observed differences. However, the longer TR employed at 3.0T acquisition should compensate for the effects caused by longer T1 at 3.0T. Also, it has been reported that T1 changes with field strength increases are less prominent.^15,22-25^

In the particular case of mI, the analysis is more complicated due to its coupled resonance, around 3.56 ppm. The mI signal arises from six CH groups which generate a complex spectral pattern and are responsible for its intrinsic low SNR in the proton MRS. In addition, its spectra overlaps with a number of other brain metabolites, including Cho, Glx, glycine (Gly), taurine (Tau), and macromolecules,^26^ which introduce uncertainty in the estimation process and increase the within-subject variability.^27,28^ As the field strength increases, the higher spectral resolution allows better separation of the mI resonances. As the relaxation time of the overlapping metabolites changes, the appearance of the mI spectra also changes at different field strengths. Thus, an accurate estimation of mI concentration requires the quantification of all of its resonances, which should be less challenging at higher field strengths. Indeed, the CRLB of mI at 3.0T are lower than at 1.5T.

The overall smaller CRLB obtained in the estimation of the metabolite concentrations at 3.0T demonstrate an important advantage of working at a higher magnetic field. This likely reflects the positive effects of the higher SNR and spectral resolution accomplished at higher fields. In addition, there was a trend for smaller variations between subjects in the metabolite quantification at higher field strength. This is also an interesting factor for clinical applications, in which pathological thresholds are established on the basis of group analyses.

The present study was performed using systems with equivalent implementation of PRESS pulse sequence and equivalent head coils. This is an advantage when compared to previous studies^10^ because accurate reproducibility of spectroscopic data depends on the efficiency of the pulse sequence used for spatial localization. This is especially important when comparing spectroscopic data acquired at different field strengths. In spite of this, small effects caused by differences in the individual optimization phase of the sequence parameters, such as water suppression and flip angle calibration, cannot be ruled out.

In conclusion, even though the theoretically predicted 100% improvement in SNR and spectral resolution cannot be achieved in practice, the benefits of higher field strengths for MRS are clear. However, due to the number of factors that can bias comparisons between field strengths, further quantitative studies at 3.0T are needed in order to redefine the normal statistical threshold for different metabolites and brain locations. Such normative studies will be crucial to improve the value of MRS as a clinical tool for diagnosis and follow-up of several neurological and psychiatric disorders.
